# Supplementary material for: Printing photonic-based thermal barrier coatings onto metal alloy
Source: Nat Commun. 2025 Jul 1;16:6034. doi: 10.1038/s41467-025-61124-2 (PMC12215888; doi:10.1038/s41467-025-61124-2)
Supplement: Supplementary file 1 — Supplementary information [file 41467_2025_61124_MOESM1_ESM.pdf]

SUPPLEMENTARY INFORMATION OF

Printing photonic-based thermal barrier coatings onto metal alloy

Alberto Gomez-Gomez[1,2,‡], Diego Ribas Gomes[3,‡], Benedikt F. Winhard[1], Laura G. Maragno[1], Antoine E. Jimenez[3], Marie Thibaudet[1], Julia Brandt[4], Alexander Petrov[4,5], Manfred Eich[4,5], and Kaline P. Furlan[3,*]

1 Hamburg University of Technology, Integrated Ceramic-based Materials Systems Group, 21073 Hamburg, Germany.
2 NETZSCH-Gerätebau GmbH, 95100 Selb, Germany.
3 Karlsruhe Institute of Technology (KIT), Institute for Applied Materials – Ceramic Materials and Technologies, Karlsruhe 76131, Germany.
4 Hamburg University of Technology, Institute of Optical and Electronic Materials, 21073 Hamburg, Germany.
5 Helmholtz Zentrum Hereon, Institute of Functional Materials for Sustainability, 21502 Geesthacht, Germany.

* Corresponding author: Prof. Kaline P. Furlan, kaline.furlan@kit.edu

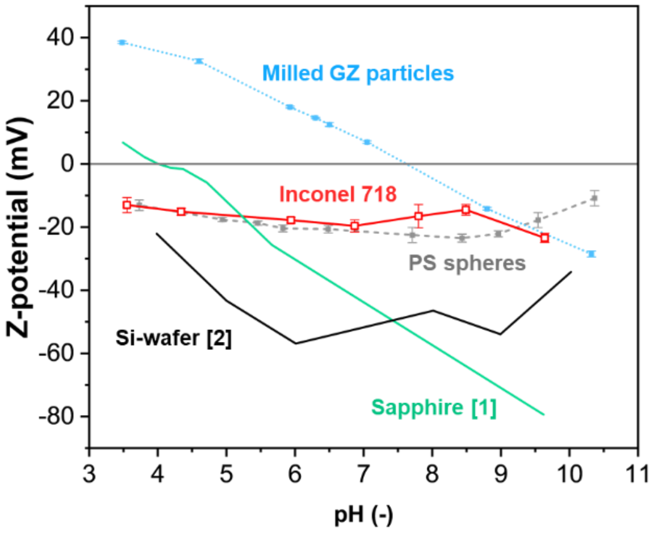

Supplementary Fig. 1 Zeta-potential of the particles used in this work ($Gd_2Zr_2O_7$ and polystyrene) and the Inconel 718, sapphire [1], and Si-wafer substrates [2]. For the Inconel substrate, polystyrene and milled gadolinium zirconate particles, each point represents the average of the measurements (3 in total per point) +/- standard deviation. Source data are provided as a Source Data file.

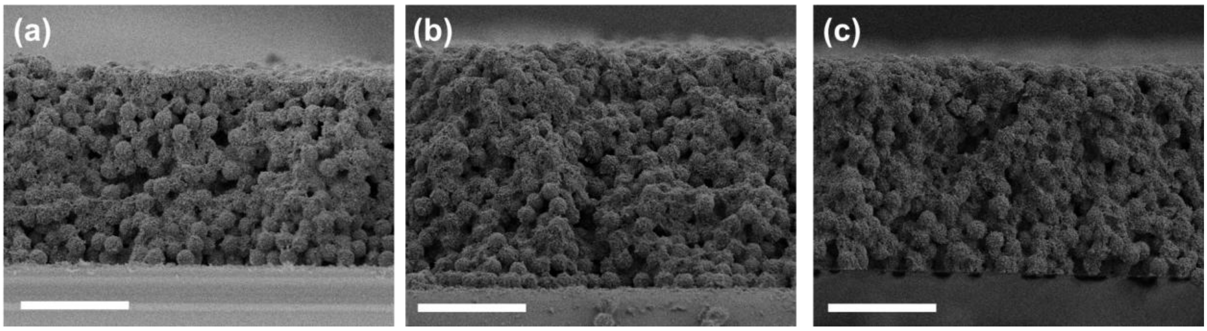

Supplementary Fig. 2 Cross section of GZ-PS PhG as-deposited on negatively-charged sapphire (a) and silicon substrates(b), as well as on glass substrates (c). Scale bar corresponds to 20 µm.

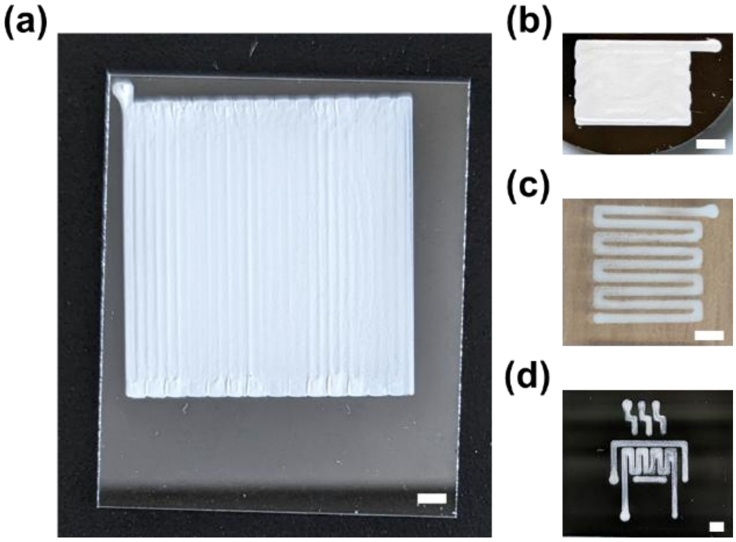

Supplementary Fig. 3 Pictures of the PhG-based coatings and patterns printed on sapphire (a), Inconel 718 (b), glass (c), and Si-wafers (d). Scale bars correspond to 2 mm.

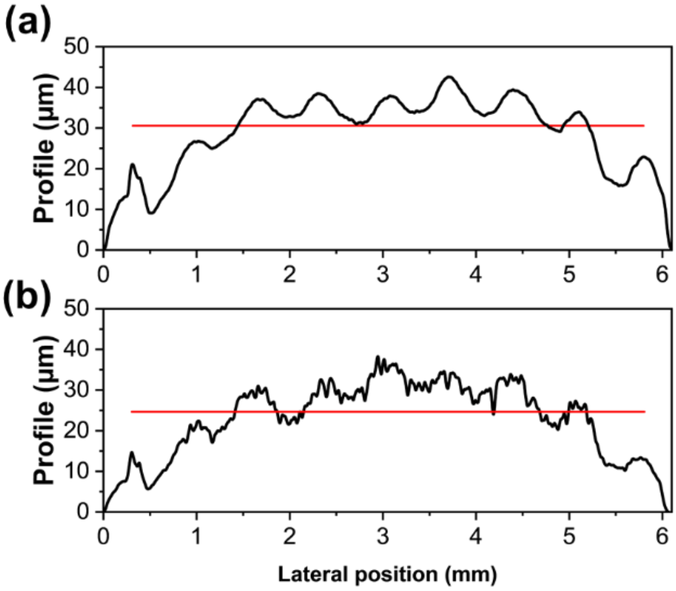

Supplementary Fig. 4 Coating profiles, acquired along transverse axis of main printing direction, deposited on horizontally placed planar Inconel 718 substrates: as deposited GZ-PS PhGs (a), and GZ-PhGs after polymeric template removal (b). Source data are provided as a Source Data file.

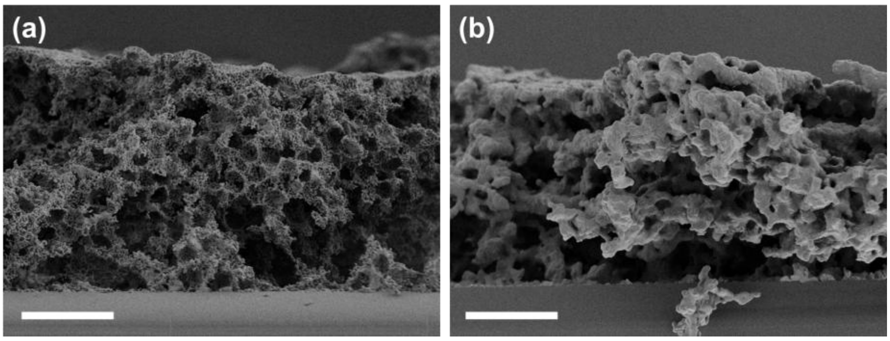

Supplementary Fig. 5 Cross section of inverse GZ PhG after 4 h at 1200 °C (a) and 4 h at 1400 °C (b). Scale bar corresponds to 10 µm.

Supplementary Table 1 Data summary of the coatings used in the reflectivity comparison.

**Coating**	**Heat treatment**	**Thickness (µm)**	**Relative density (%)**	**Substrate material**
GZ “inverse” PhG	1200 °C – 4 h	21	35.0†	Sapphire
GZ “inverse” PhG	1400 °C – 4 h	21	35.0†	Sapphire
Mullite “inverse” PhGs [5]	1500 °C – 24 h	20	45.1‡	Sapphire
YSZ “inverse” PhG [7]	1200 °C – 120 h	32	35.0†	Sapphire
YSZ direct PhG [43]	1200 °C – 192 h	100	54.9‡	Sapphire
YSZ TBC (APS) [41]	-	200	74.7	Free-standing coating
GZ TBC (APS) [42]	800 °C – 4 h	110	85.4	Free-standing coating
YSZ TBC (EB-PVD) [40]	950 °C – 20 h	122	80.0ǀ	Rene N5 (Ni-based superalloy)
Multilayered YSZ TBC (EB-PVD) [40]	950 °C – 20 h	133	80.0ǀ	Rene N5 (Ni-based superalloy)

†Relative density estimated based on results of Ptychographic X-ray Computed Tomography (PXCT) analysis of YSZ-PhG structures produced by a similar route as the one used in this study [44].

‡Relative density calculated from the filling fraction of PhGs estimated via X-ray nanotomography by Ogurreck *et al.* [45].

ǀRelative density calculated from the porosity values of EB-PVD TBCs obtained via Small-angle X-ray scattering (SAXS) by Kulkarni *et al* [46].

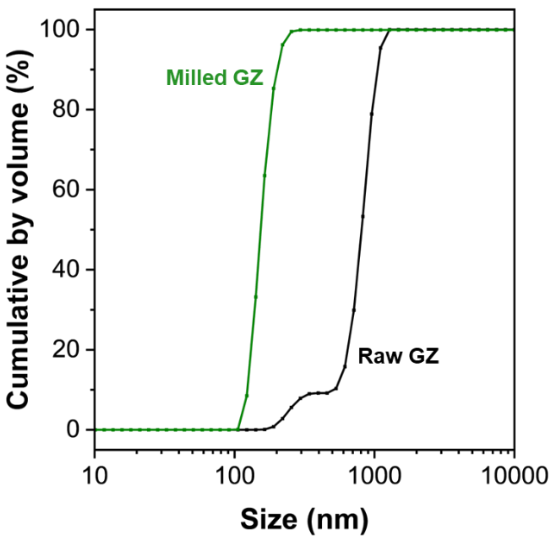

Supplementary Fig. 6 Size distribution of Gd2Zr2O7 (GZ) particles before and after planetary milling. Values for d10, d50 and d90 are provided in Table S1. Source data are provided as a Source Data file.

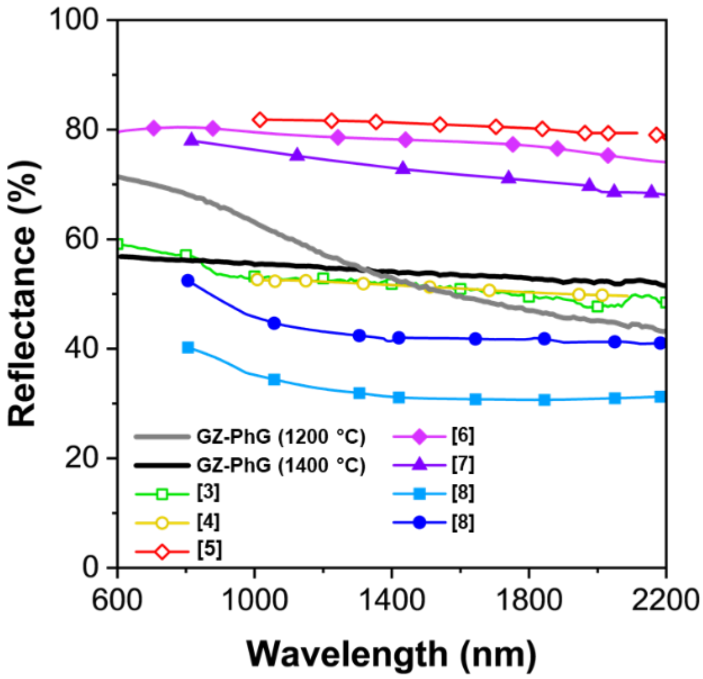

Supplementary Fig. 7 Reflection spectra of GZ PhGs, mullite PhGs [3], YSZ inverse PhGs [4], YSZ direct PhGs [5], YSZ-TBCs produced by APS [6], GZ-TBCs produced by APS [7], YSZ-TBCs produced by EB-PVD [8]. The hemispherical reflection spectra of GZ PhGs from 600 nm to 2200 nm wavelength were obtained using a UV–Vis-NIR spectrometer (Lambda 1050 equipped with an integrating sphere accessory, Perkin-Elmer). All photonic structures in this comparison were “inverse” PhGs, except the YSZ PhG (represented with red diamonds), which were a direct PhG. EB-PVD-produced YSZ-TBC represented with blue circles consisted of a multilayered structure for optimising the NIR reflectivity. The squares, circles, triangles and diamonds on the experimental measurements of the reflectivity of the various TBCs used in the comparison serve only to guide the eye. Source data are provided as a Source Data file.

Supplementary Table 2 Summary of percentiles (d10, d50 and d90) for the particle size distributions of raw and milled GZ.

Sample	d_{10} (nm)	d_{50} (nm)	d_{90} (nm)
$Gd_2Zr_2O_7$ raw powder	538	870	1150
Milled $Gd_2Zr_2O_7$ powder	129	166	219

Supplementary Table 3 Summary of the printing parameters for the deposition of GZ-PS PhGs onto different substrates.

		**Planar**						**Curved**
		**Inconel**		**Sapphire**		**Silicon**	**Glass**	**Inconel**
Inclination (°)		0	23	0	23	0	0	-
Writing velocity ($mm \cdot s^{-1}$)	1st comb	1	1	1	1	1	1	1
	2nd comb	2	2	2	2	2	2	2
	3rd comb	-	2	-	2	-	-	2
Dispense velocity ($\mu m \cdot s^{-1}$)	1st comb	127	127	127	127	127	127	253
	2nd comb	950	950	950	950	2533	1900	1076
	3rd comb	-	950	-	950	-	-	1393
Distance between lines (mm)		0.7	0.7	0.7	0.7	1.0	1.0	0.7

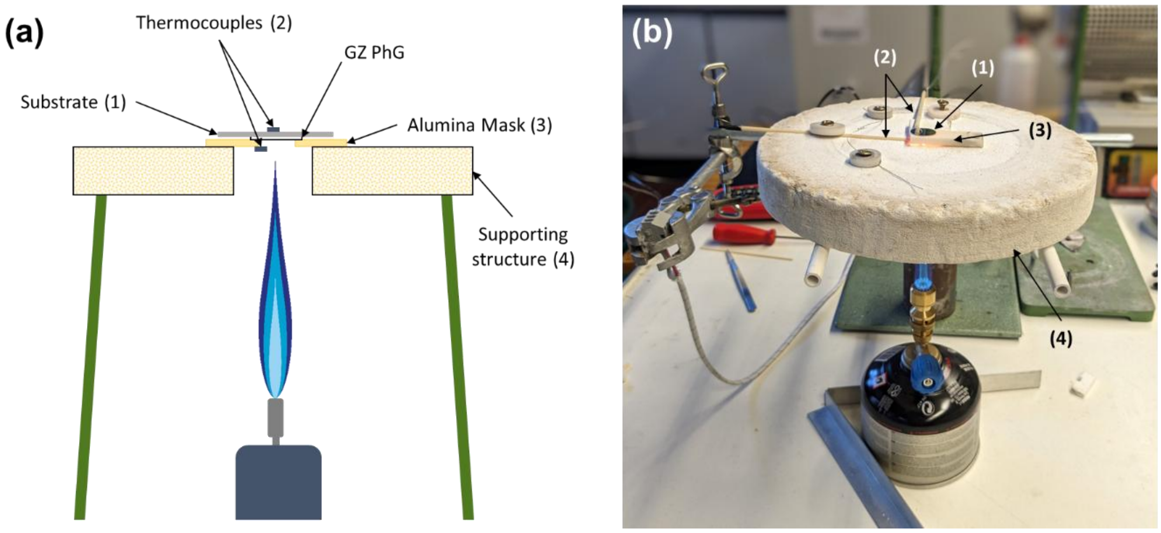

Supplementary Fig. 8 (a) Schematic representation of the torch experimental setup and (b) digital photograph of the device during the experiments.

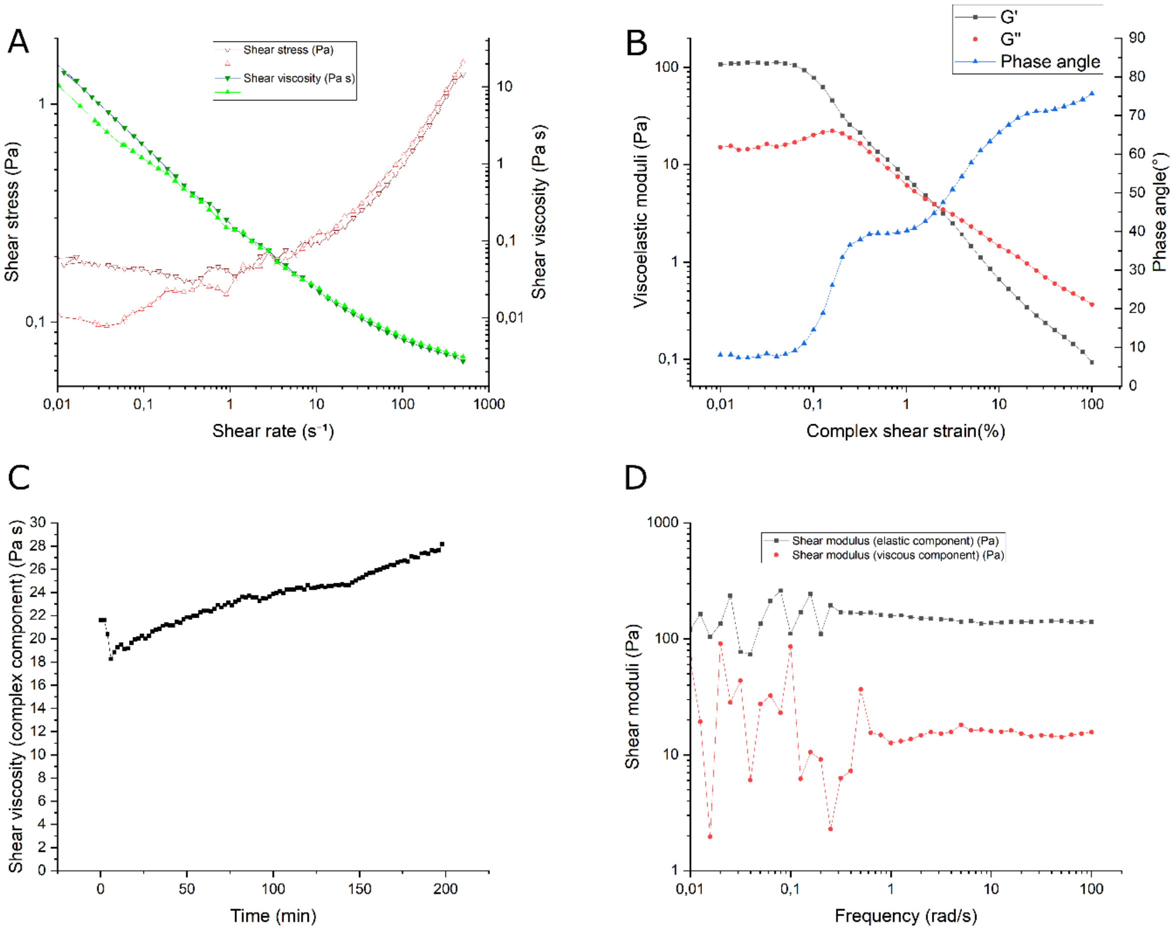

Supplementary Fig. 9 Rheological properties of the ink used for AMCA printing. (A) Flow curve showing shear-thinning behaviour with up-facing markers indicating increasing shear rate and down-facing markers indicating decreasing shear rate; (B) Amplitude sweep revealing a linear viscoelastic region (LVER) up to ~0.05% strain (0.07 Pa), with a flow point at ~2% strain (0.12 Pa) (C) Time-resolved oscillatory measurement (0.03% strain) showing a gradual viscosity increase from 21 to 28 Pa·s over 3 hours; (D) Frequency sweep (performed at 0.03% strain, within the LVER) demonstrating a stable elastic modulus (G' ~140 Pa) and viscous modulus (G" ~16 Pa) over the tested frequency range (0.01–100 rad/s). Source data are provided as a Source Data file.

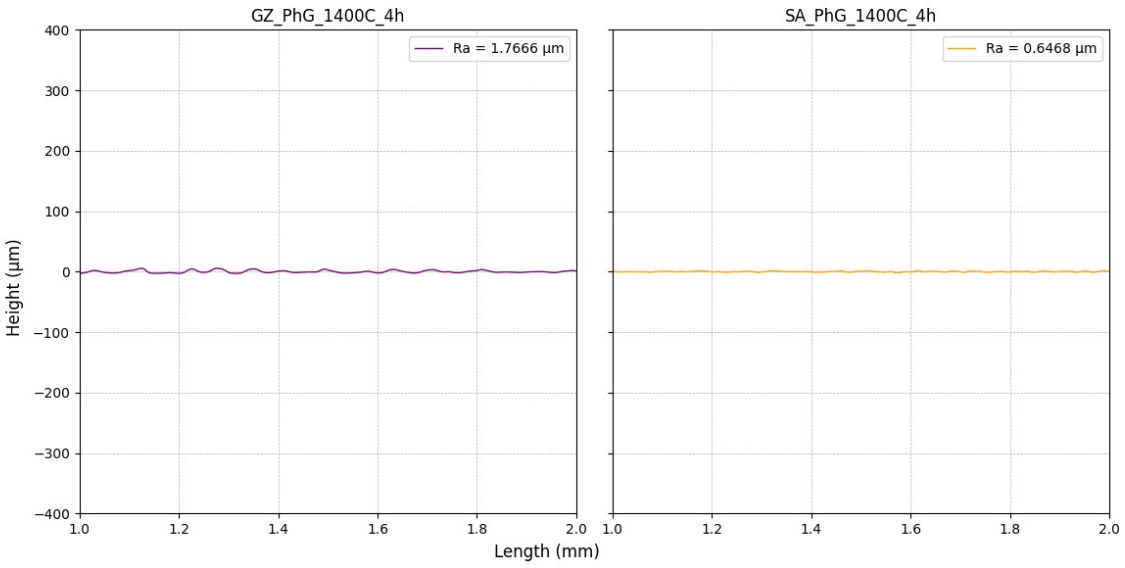

Supplementary Fig. 10 Optical profilometry of GZ (left) and sol-gel-based mullite (right) PhGs. Source data are provided as a Source Data file.

References used in this file

1. Häntsch QY (2021) Colloidal self-assembly – from controlled deposition to structural colors. Ph.D. thesis. University: Technische Universität Hamburg. Dr. Hut, Hamburg, Germany. ISBN: 978-3-8439-4970-5.

2. Itano M, Kezuka T, Ishii M, Unemoto T, Kubo M, Ohmi T (1995) Minimization of Particle Contamination during Wet Processing of Si Wafers. J Electrochem Soc 142:971-978. https://doi.org/10.1149/1.2048570

3. Gomez-Gomez A, Gomes DR, Winhard BF, Maragno LG, Krekeler T, Ritter M, Furlan KP (2023) Mullite photonic glasses with exceptional thermal stability for novel reflective thermal barrier coatings. J Mater Sci 58:12993–13008. https://doi.org/10.1007/s10853-023-08844-2

4. Do Rosário JJ, Häntsch Y, Pasquarelli RM, Dyachenko PN, Vriend E, Petrov AY, Furlan KP, Eich M, Schneider GA (2019) Advancing the fabrication of YSZ-inverse photonic glasses for broadband omnidirectional reflector films. J Eur Ceram Soc 39:3353-3363. https://doi.org/10.1016/j.jeurceramsoc.2019.04.028

5. Leib EW, Pasquarelli RM, Do Rosário JJ, Dyachenko PN, Doering S, Puchert A, Petrov AY, Eich M, Schneider GA, Janssen R, Weller H, Vossmeyer T (2016) Yttria-stabilized zirconia microspheres: novel building blocks for high-temperature photonics. J Mater Chem C 4:62–74. https://doi.org/10.1039/C5TC03260A

6. Yang G, Zhao CY (2015) A comparative experimental study on radiative properties of EB-PVD and air plasma sprayed thermal barrier coatings. J Heat Transf 137:091024. https://doi.org/10.1115/1.4030243

7. Wang L, Eldridge JI, Guo SM (2013) Thermal radiation properties of plasma-sprayed Gd2Zr2O7 thermal barrier coatings. Scr Mater 69:674–677. https://doi.org/10.1016/j.scriptamat.2013.07.026

8. Kelly MJ, Wolfe DE, Singh J, Eldridge J, Zhu DM, Miller R (2006) Thermal barrier coatings design with increased reflectivity and lower thermal conductivity for high-temperature turbine applications. Int J Appl Ceram Techn 3:81–93. https://doi.org/10.1111/j.1744-7402.2006.02073.x
